# Preliminary study on the spread of air-borne pollutants in urban environment: a CFD simulation approach

**DOI:** 10.1038/s41598-025-03197-z

**Published:** 2025-05-29

**Authors:** Fatma Ahmad, Debjit Majumder, Rabs Ranjit, Aman Gupta, Michael Manhart

**Affiliations:** 1 Chair of Computing in Civil Engineering and Geoinformation Systems and Geodetic Institute, RWTH Aachen University, Aachen, Germany; 2 School of Engineering and Design, Technical University of Munich, Munich, Germany; 3 Institute of Geography, School of Geosciences, University of Edinburgh, Edinburgh, UK; 4 Department of Architecture and Planning, Indian Institute of Engineering Science and Technology, Shibpur, India

**Keywords:** Air quality, Computational fluid dynamics, Large eddy simulation, Wind simulation, Environmental sciences, Engineering

## Abstract

The spreading of pollutants within urban areas, particularly from traffic emissions, poses a significant health risk. Computational Fluid Dynamics (CFD) has emerged as a key tool in understanding how pollutants spread within a city. In particular, the large-eddy simulation (LES) approach allows us to capture the complex time-dependent behaviour of the 3D flow field due to buildings in a dense urban environment. This work utilizes the CFD tool MGLET (Multi Grid Large Eddy Turbulence) to model the transport of pollutants within a selected domain in Munich city. MGLET offers a feature of simulating transport of multiple passive scalar quantities simultaneously. This facilitates the individual analysis of emissions from each major street in the domain of interest, providing detailed insights into their respective impacts. Additionally, MGLET utilizes the Immersed Boundary Method to resolve 3D building geometries, removing the need to generate body-fitted grids, which tends to be highly time-consuming. The streets are defined by area sources, and the emission rates for each street are defined by the average traffic flow rate. This high-fidelity approach offers a detailed analysis, allowing us to identify local features such as recirculation zones in street canyons and pinpoint the streets that contribute most significantly to pollution at specific locations. The insights from CFD studies can empower policymakers to craft legislation tailored to local pollution control efforts, thus enhancing the quality of life in urban areas. Ultimately, the accurate prediction of pollutant concentration is critical, as it directly impacts the health and well-being of urban residents, highlighting the urgent need for effective pollution control measures.

## Introduction

The global increase in vehicular emissions and declining air quality have become critical concerns, highlighting the need for advanced research into air pollution and its health impacts^1,2^. The transport sector contributes significantly to global air pollution, accounting for approximately 33% of transportation-related CO₂ emissions in 2020^3^. In developed nations, such as OECD countries (Organisation for Economic Co-operation and Development), advances in vehicle technology and fuel quality have seen a successful reduction in vehicular emissions since 1990 despite increasing travel patterns^4^. Regionally, road transport contributes 20% of PM2.5 emissions in the EU and 55% of NOx emissions in the US. However, in contrast, many developing countries are experiencing rapid increases in vehicle emissions due to growing vehicle fleets and less stringent emission standards. In developing nations, transport is responsible for around 70% of urban air pollution. Globally, outdoor air pollution causes 4.2 million premature deaths annually^5^.

In response to these agencies, researchers worldwide have begun employing advanced tools to model emissions and air quality^6^. While statistical modelling has been widely used, it has limitations, particularly in its inability to explicitly represent airflow and turbulence^7^. In order to address these shortcomings, computational fluid dynamics (CFD) methods are increasingly being used to model airflow and turbulent eddies^8,9^. Several best practice guidelines have been proposed to ensure the reliability of CFD simulations in urban environments^10^.

High population densities in cities increase exposure to air pollutants, making effective air quality monitoring crucial^11^. However, the dense urban infrastructure creates turbulent airflows, complicating the study of fluid dynamics and the calculation of pollutant concentration and dispersion using traditional methods^12,13^. The CFD approach for the scope of this study is done using the incompressible Navier-Stokes equations flow solver MGLET (Multi Grid Large Eddy Turbulence)^14,15^. Because of the computational expense of direct numerical simulation (DNS)^16^, wind simulation along the region of study is done with large eddy simulation (LES), making it computationally feasible for turbulence modelling.

The study focuses on the transport of pollutants emitted from the city’s street network by wind. A case study assumes a steady wind profile originating from the west side of the domain, with data sourced from the German Weather Service^17^. Hourly resolution data from 2020 were used, with measurements taken at the Munich Airport station. Given the westward flow, the domain is designed to reflect a fully developed atmospheric boundary layer (ABL) with a length-to-width ratio that is longer than wide. In the Munich region, the domain dimensions are (3,900 m × 1,900 m × 1,000 m). Additionally, the domain extends up to 1,000 m above mean sea level in the vertical direction. This design ensures that the ABL is accurately represented without constraining important turbulent structures and allows for the simulation of the surrounding terrain^18^.

Among all the available air quality stations within Munich city, Karlsplatz-Stachus is considered the benchmark station in this scope, as it is located in a dense urban infrastructure. This modelling approach correlates weather variables, like wind speed and wind direction, with air pollution concentrations. To support emissions modelling, a conversion scaling factor was applied to translate pollutant concentrations (µg/m³) into estimated emission rates (g/day). This was essential for simulating traffic-related NOx emissions across the study grid. Additionally, a calibration approach was used in the inverse direction to estimate NOx concentrations from known traffic emissions. A reference station (Stachus) provided a concentration-to-emission ratio, which was then applied to other locations. This method was validated against two other stations, showing good agreement and confirming the reliability of the emission-concentration linkage (Table 1). QGIS was employed to map the street network and create a georeferenced point cloud, which was overlaid on building geometries and a topographic standard tessellation language (STL) map of Munich.

Although the MGLET model requires further refinement for industrial-scale applications such as high-resolution simulations across large urban-industrial zones or integration with real-time emission monitoring systems, it demonstrates significant potential to advance Sustainable Development Goals (SDGs)^19^, particularly SDG 11 (Sustainable Cities and Communities) and SDG 13 (Climate Action). By simulating urban pollutant transport, the model provides policymakers, urban planners, and environmental scientists with critical insights for designing resilient cities with improved air quality. Its data-driven approach enables targeted pollution control strategies, supporting evidence-based decision-making. With further development, MGLET could also guide industries in adopting cleaner technologies, optimizing emissions reductions, and minimizing environmental impacts—ultimately enhancing quality of life and accelerating sustainable urban development.

**Table 1:** Comparison between NOx concentration values calculated with the calibration method and values measured at two other stations.

	Measured concentration value (Ug/m^3^)	Expected concentration value (Ug/m^3^)	Difference %
NOx total Year Landhutte alle—Cell 4.2 (Ug/m^3^) 11 Nov	54.385	54.385	CALIB.
Traffic NOx total Year Landhutte alle—Cell 4.2 (Ug/m^3^)	22.31179487		CALIB.
NOx total Year Stachus—Cell 5.4 (Ug/m^3^) 11 Nov	33.465		
Traffic NOx total Year Stachus—Cell 4.2 (Ug/m^3^)	13.72923077	12.36	− 9.99
NOx total Year Lohnstrasse—Cell 3.3 (Ug/m^3^) 11 Nov	23.13		
Traffic NOx total Year Lohnstrasse—Cell 4.2 (Ug/m^3^)	9.489230769	9.37	− 1.24

## Overview on multi grid large eddy turbulence (MGLET)

MGLET^20^ is a CFD software package written in FORTRAN that has been developed and maintained at the Professorship of Hydromechanics, Technical University of Munich, Germany. It stands for Multi Grid Large Eddy Turbulence. The code was originally developed in the 1980s by Heinrich Werner during his PhD as a LES version of the RANS-CFD code STAR and has been continuously refined and extended since then^21^. MGLET employs a finite-volume method to solve the divergence form of the incompressible Navier-Stokes equations for the primitive variables (i.e., three velocity components and pressure) in order to simulate complex flow phenomena within an arbitrarily shaped domain. The time integration is done by an explicit third-order low-storage Runge-Kutta scheme. The pressure computation is decoupled from the velocity computation by Chorin’s projection method. That is, a Poisson equation is solved for the pressure for each Runge-Kutta sub-step. The code is capable of performing DNS and LES of complex turbulent flows, which can be optionally coupled with the transport of some scalar quantities using the Advection-Diffusion equation. This capability of MGLET to couple turbulence models with scalar transport is of particular interest to this project. For LES, MGLET offers Smagorinsky and WALE (Wall-Adapting Local Eddy-Viscosity) models for subgrid-scale turbulence modelling^22,23^. Arbitrarily curved and geometrically complex surfaces in MGLET are handled by an immersed boundary method^24^, which allows us to simply introduce the obstacles, such as terrain and buildings, as STL files within the domain. MGLET also allows the parallelization of computation for optimal performance and accelerated simulations. The grid generation of the domain for MGLET is done by Python grid generator ’gridgen3’, which is currently under development at TUM. It is able to create multi-level 3D grids with the provision of local refinements, which allows high spatial resolution computation in regions of interest. The boundary conditions of the domain are also defined within the gridgen3 python script. The results from the simulation can then be post-processed in ParaView-5.13.0^25^, an open-source data analysis and visualization application.

## Research objectives

The primary objective of this study is to capture the dynamics of pollutant transport and dispersion in urban environments using the MGLET software. By simulating the flow of pollutants from traffic emissions and their interactions with urban structures, the study aims to provide valuable insights into how urban design, traffic patterns, and atmospheric conditions contribute to air quality in cities. The specific objectives are as follows:

1. To capture the key features of urban airflow, such as wind profile and recirculation, in order to accurately model the dispersion and concentration of pollutants.
2. To analyse the contributions of different street networks and traffic flow patterns to the distribution of air pollutants, with a focus on how these factors influence local air quality.
3. To identify areas for future improvements in the model, focusing on enhancing accuracy in simulating complex urban geometries, meteorological effects, and present pollutant levels and expanding its applicability to industrial-scale scenarios.

**Methods**.

### Meteorological and traffic data

LES has been extensively used in urban flow studies due to its ability to capture complex turbulent structures^26–32^. The final objective of this study is to perform an LES using MGLET to track the spread of air-borne pollutants in the urban environment of Munich. Additionally, we aim to calibrate the model with respect to concentration data from the air-quality measuring station at Karlsplatz Stachus. The methodology focuses on selecting and integrating datasets to simulate traffic-induced pollutant emissions in Munich, ensuring temporal, spatial, and contextual compatibility. Data selection was guided by specific criteria prioritizing free data sources, reliability from official authorities, and temporal coverage of at least five years. A single year, 2020, was chosen for its data availability at hourly resolution. This year offered the most comprehensive and representative dataset for Munich, particularly its central areas where monitoring stations and the STL model of the city’s topography and buildings are concentrated.

Air quality concentration data were obtained from the Bavarian State Office for the Environment^33^, which operates high-quality monitoring stations across Bavaria, including five in Munich that met the study’s resolution and central coverage requirements. While the Municipality of Munich listed 44 stations, their data lacked the required resolution. Hourly data for these five stations were tabulated, and trends were analysed for the entire year. Climatic data, including wind speed, wind direction, and ambient temperature, were sourced from the German Weather Service (*Deutscher Wetterdienst*), with measurements from the Munich Airport station providing the necessary metadata.

Traffic data were acquired from the Mobility Department of the City of Munich, State Capital Munich Mobility Department^34^, presented as maps depicting average daily traffic volumes for a typical day in 2020. These maps were digitized into a 64-cell grid using AutoCAD, with traffic volumes weighted by street influence. Road lengths and traffic volumes within each cell were estimated for major road segments to ensure representativeness. The total NOx emissions for each cell were calculated using the following formula^35^:

${\text{NOx }}\left( {{\text{g}}/{\text{day}}} \right)\,=\,{\text{Road Length }}\left( {{\text{km}}} \right) \times {\text{Traffic Volume }}\left( {{\text{pkW}}/{\text{day}}} \right) \times {\text{Emission Factors}}$where: pkW/day (Personenkraftwagen-Einheiten pro Kilometer): Passenger car units per kilometer per day.

Emission factors were derived from European Union standards for passenger cars (Category M, Euro 6d Regulation) and heavy-duty vehicles (Euro VI). For heavy-duty vehicles, conversion factors between g/kWh and g/day were applied, as recommended by regulatory sources^36^. It accounts for typical energy consumption rates and operational patterns of commercial vehicles.

The model calibration process involved initial validation by comparing computed NOx concentrations with actual measurements at a primary monitoring station, followed by subsequent verification using data from two additional stations to confirm the proportional relationships between estimated and observed pollution levels. To achieve hourly resolution in the traffic data, we incorporated mobility patterns derived from mobile network analyses^37^. These temporal distribution patterns, obtained from anonymized vehicle movement data, were represented as histogram-based distribution curves and applied to proportionally allocate daily traffic volumes across 24-hour periods, enabling time-sensitive emission modelling that reflects real-world traffic fluctuations.

### OpenStreetMap data for the region of interest

In order to develop a comprehensive model of the road network within the study area, data were sourced from OpenStreetMap (OSM) using the OSM Plugin in QGIS and georeferenced with the WGS84 coordinate system. A line layer representing roads and pathways was generated through the ‘Vector’ tool, complemented by a point layer marking the vertices of these geometries. These spatial datasets facilitated the creation of three-dimensional (3D) models of the region’s topography and built environment, represented as STL files comprising triangular meshes. The STL files, visualized using ParaView, retained simplified coordinates corresponding to the real-world geographic positions of vertices, ensuring spatial accuracy.

The topography of the study area was represented using STL (stereolithography) files composed of small triangular facets approximating real-world surfaces. To isolate emission-relevant geometry, a Python-based method was developed to relate QGIS-generated street lines with corresponding STL surface triangles.

**Fig. 1:**
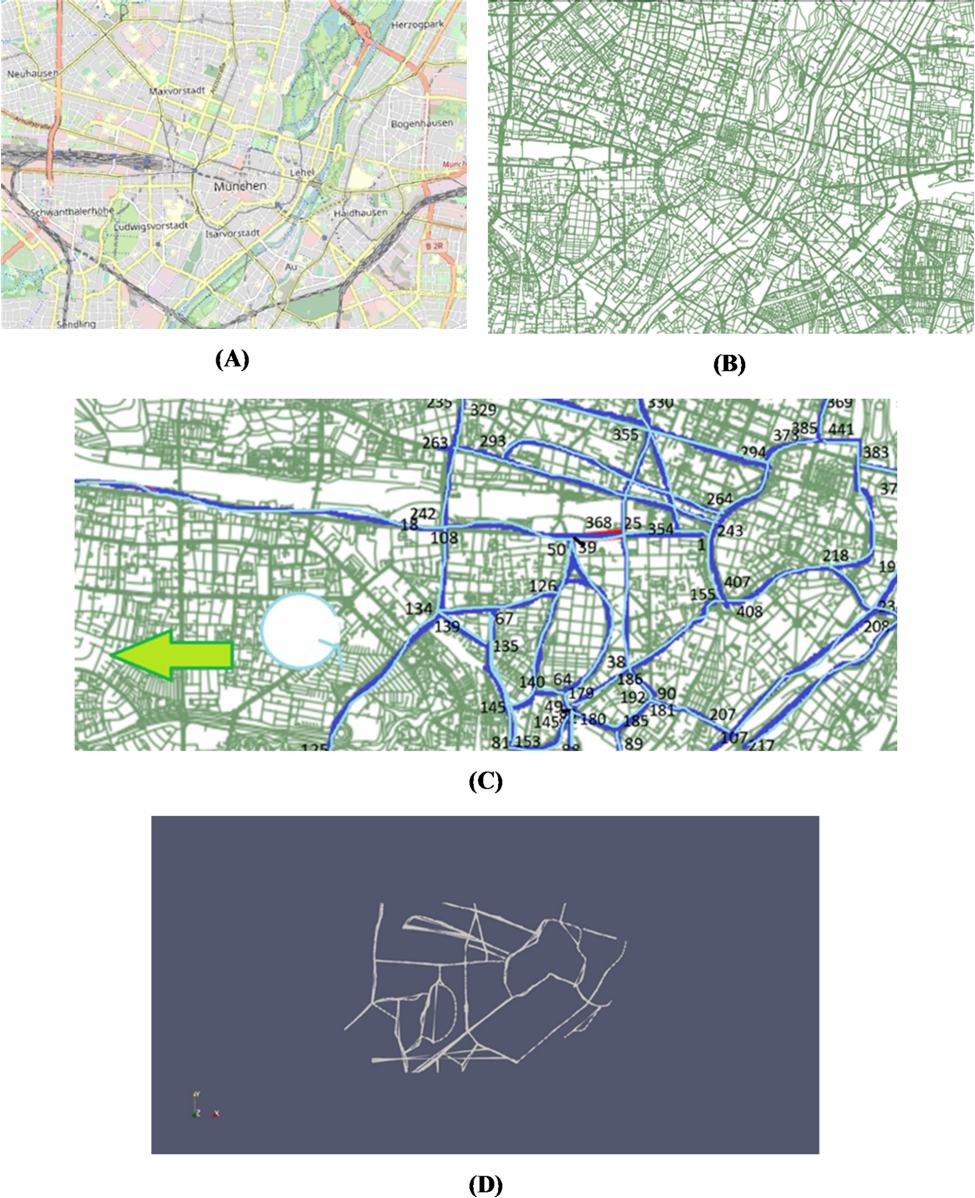
Python-based methodology to isolate and analyse specific streets of interest (Imagery sourced from OpenStreetMap, https://www.openstreetmap.org/ and processed in QGIS, https://qgis.org/). (**A**) OSM view in QGIS. (**B**) Line-Layer view in QGIS. (**C**) Subnetwork of selected pathways for Python code development. (**D**) Extracted Street subnetwork using Python code.

First, a subset of streets of interest was selected based on unique street IDs (see Fig. 1). For each selected street, a centreline was constructed using its start and end nodes. A rectangular buffer zone extending 15 m on either side of this centreline was created to approximate the full width of the roadway, sidewalks, and adjacent vehicle-operating areas—aligning with typical urban street widths in Munich (10–30 m total)^38,39^.

Using polygon intersection techniques, the routine identified STL triangles that overlapped with each buffer zone. Each triangle within this defined domain was tested for intersection, and those overlapping were extracted as representing the street’s physical surface. The routine was parallelized using mpirun across 18 processes, resulting in the successful extraction of STL triangles corresponding to 58 streets in and around the Stachus area.

### Domain selection

The selection of an appropriate domain size is critical to ensure the development of a fully established atmospheric boundary layer and to mitigate recirculation effects. The vertical expansion of the domain is guided by the methodology outlined in the work of^18^. From the georeferenced STL files generated in the previous step, a domain measuring 3900 × 3900 × 1000 units was selected from the total extracted area of Munich for further analysis.

### Inflow wind profile

To incorporate a more realistic scenario, we adhered to the guidelines outlined in^40^. These guidelines classify urban areas as Category-IV terrain, which prescribes a relationship between mean wind speed and height above the ground, as described in Eq. 1. Furthermore, we aimed to align the velocity profile with the wind data recorded at the Munich Airport wind station. The Munich Airport wind station’s sensor is positioned at an elevation of 453 m above mean sea level and 8 m above the ground.

1 $$\:{v}_{m}=0.56\times\:{v}_{b}\times\:\:{\left(\frac{z}{10}\right)}^{\alpha\:}$$

$$\:\text{w}\text{h}\text{e}\text{r}\text{e},\:{v}_{m}=mean\:velocity\:\left[\frac{m}{s}\right];\:{v}_{b}=base\:velocity\:\left[\frac{m}{s}\right];\:{v}_{m}=height\:above\:ground\:level\:\left[m\right];\:\alpha\:=-0.30\:for\:urban\:environment.$$

To achieve a better alignment between the wind speed measured by the station and the simulated wind speed, we introduced a calibration factor to the recommended equation. This deviation allowed us to adjust the wind speed at a height of 8 m above the terrain’s westernmost side while preserving the power-law wind profile. The elevation range of the westernmost side of the domain spans from 522 m to 540 m. Based on this information, we made an assumption that wind speeds at elevations lower than the reference elevation (assumed to be 532 m) follow a block wind profile equal to a quarter of the measured wind speed. Above the reference elevation, the base block wind profile is augmented with a power-law profile. These assumptions can be seen in Eq. (2).

$$\:{\text{v}}_{\text{m}\text{e}\text{a}\text{n}}=\left\{\begin{array}{c}\frac{{\text{v}\text{b}}_{\text{s}\text{e}\text{n}\text{s}\text{o}\text{r}}}{4}\:\:\:\:\:\:\:\:\:\:\:\:\:\:\:\:\:\:\:\:\:\:\:\:\:\:\:\:\:\:\:\:\:\:\:\:\:\:\:\:\:\:\:\:\:\:\:\:\:\:\:\:\:\:\:\:\:\:\:\:\:\:\:\:\:\:\:\:\:\:\:\:\:\:\:\:\:\:\:\:\:\:\:\:\:\:\:\:\:\:\:\:\:\:for\:elev.\le\:ref.\:elev.\\\:\left(\frac{{\text{v}\text{b}}_{\text{s}\text{e}\text{n}\text{s}\text{o}\text{r}}}{4}\right)+Calibration\times\:0.56\times\:{\text{v}\text{b}}_{\text{s}\text{e}\text{n}\text{s}\text{o}\text{r}}\times\:{\left(\frac{\left(\text{e}\text{l}\text{e}\text{v}-\text{R}\text{e}\text{f}\text{E}\text{l}\text{e}\text{v}\right)}{10.0}\right)}^{0.3}otherwise\end{array}\right.$$

where,

2 $$\:\text{C}\text{a}\text{l}\text{i}\text{b}\text{r}\text{a}\text{t}\text{i}\text{o}\text{n}=\frac{{\text{v}\text{b}}_{\text{s}\text{e}\text{n}\text{s}\text{o}\text{r}}}{0.56\times\:{\text{v}\text{b}}_{\text{s}\text{e}\text{n}\text{s}\text{o}\text{r}}\times\:{\left(\text{S}\text{e}\text{n}\text{s}\text{o}\text{r}\text{H}\text{e}\text{i}\text{g}\text{h}\text{t}/10\right)}^{0.3}}$$

Such inflow profile calibration and turbulence synthesis strategies are critical for ensuring the accuracy of LES in urban CFD. Several methods have been reviewed and tested in prior studies^41–44^.

### Meshing, local refinements and boundary conditions

This MGLET tool is scripted to create grids with three different levels of resolution. In order to ensure an accurate representation of the street width, the finest level of the grid was designed to have a target resolution where at least three cells correspond to the width of the streets. Considering an average street width of 7.5 m, the appropriate range for the cell width would be around 2.5 m. Based on previous studies conducted with MGLET, it was observed that LES performs better when the grid cells have cubic dimensions. Therefore, the target spatial discretization was set to approximately ∆x ≈ ∆y ≈ ∆z ≈ 2.5 m. Keeping the total number of cells below 250 million was advised to maintain computational efficiency. Considering these constraints, the mesh design was carefully orchestrated to meet the desired criteria. The final arrangement of the adopted meshing can be observed in Table 2.

**Table 2:** Meshing design.

Direction	Min. range	Max. range	Domain length	No. of cells	∆x_i_ at level 3
**x**	7100	11,000	3900	1536	2.54
**y**	9100	11,000	1900	768	2.47
**z**	500	1000	500	192	2.60
Total cells = **226**,**492**,**416.00**					

While a resolution of three cells per street width may seem limited for Large-Eddy Simulation (LES), this approach follows common practices in urban-scale LES studies, balancing resolution with computational feasibility. A study^45^ showed that LES with 1 m near-wall spacing and 8.5–10 million elements performed well in urban domains, supporting our choice of cell size. While further refinement could improve accuracy, this setup meets established urban LES standards.

**Fig. 2:**
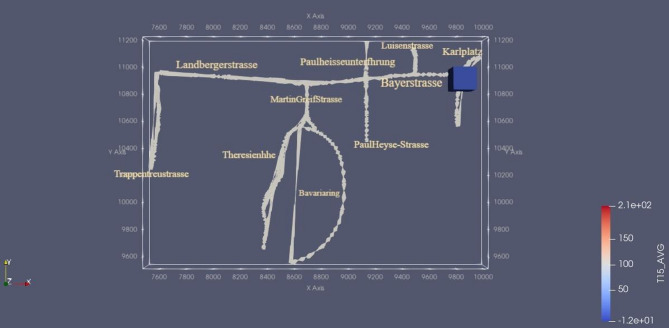
Influencing streets for emissions at Karlplatz Stachus (visualization of street on ParaView-5.13.0, https://www.paraview.org, showing scalar average concentration).

**Fig. 3:**
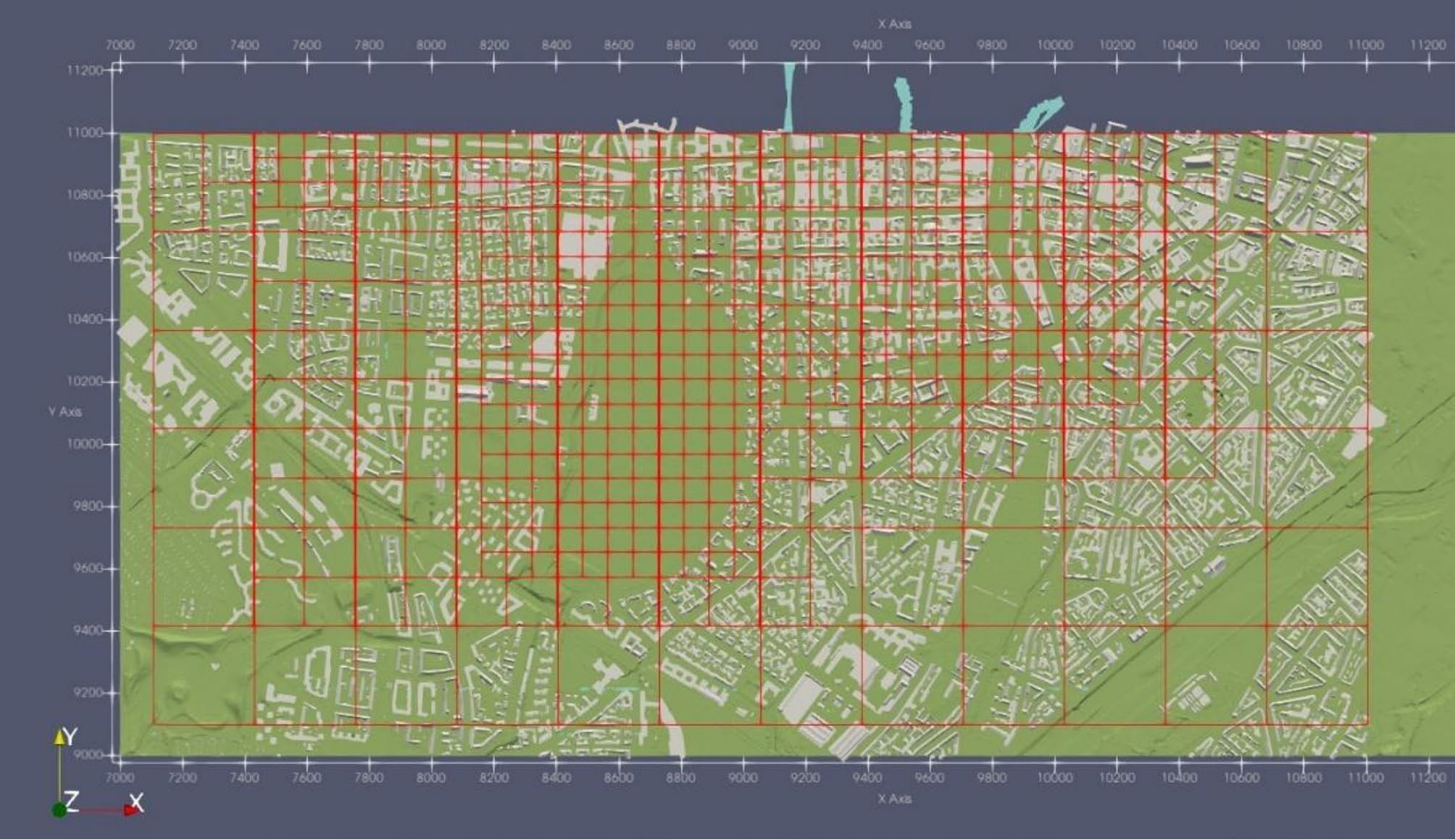
Domain extent in the west wind direction with grid resolution based on regions of interest (Test data of Munich urban terrain as STL bodies from MGLET source code visualized in ParaView-5.13.0).

**Fig. 4:**
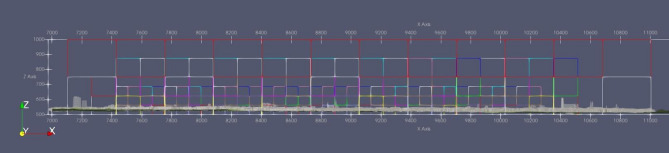
Refinement of domain in XY plain (Domain scripted in MGLET grid generator and viewed on ParaView-5.13.0, https://www.paraview.org).

Furthermore, it is crucial to identify specific regions for local refinement based on the final grid level in order to enhance the accuracy of the simulations and the efficiency of computations. One such region of interest is the air quality station located at Karlsplatz-Stachus, situated approximately at coordinates x = 10,000 m and y = 10,800 m within the domain. Preliminary simulations conducted at a coarser resolution revealed that the concentration of pollutants in this particular area is significantly influenced by the nearby streets to the west and the streets surrounding Bavariaring (Fig. 2). As a result, these specific areas were chosen for local refinement in order to capture the fine-scale dynamics accurately. In Fig. 3, the refinement areas are depicted, characterized by smaller grid boxes compared to the surrounding regions. Notably, the refinement strategy was also implemented in the z-axis, with the finest grids assigned to the elevation range of 500 m to 670 m above mean sea level. The refinement pattern in the z-axis can be observed in Fig. 4.

The boundary conditions for the domain were selected as follows:

1. Fixed boundary condition for the west side (inlet).
2. Open boundary condition for the east side (outlet).
3. No slip boundary condition for the bottom.
4. Slip boundary condition for the top, north, and south sides.

### Transport of multiple scalars

There are 34 major streets within the domain, and each street was assigned a single scalar with unit flux. Essentially, the idea was to simulate the advection-diffusion process for each scalar separately. Then, all 34 scalars would be superimposed in the post-processing, where weights are given to each scalar. The calibration of the model would then be done by altering the values of these weights such that the total concentration of pollutants matched the observed value.

## Results and observations

### Simulation in coarser grid resolution

The first runs of the simulation were on a coarser grid to save time and computational expense while testing the new patch of MGLET. These simulations were run in the GNU/Linux-based HYD37 server at the Chair of Hydromechanics. The coarser grid was set with only one grid level with ∆x ≈ ∆y ≈ ∆z ≈ 5.0 m. The simulation was run for approximately 2800 s on LES with WALE sub-grid scale modelling with custom power-law wind profile and ∆t = 0.03 s. Some of the noteworthy observations from the simulation are explained below.

#### Checker-board patterns

**Fig. 5:**
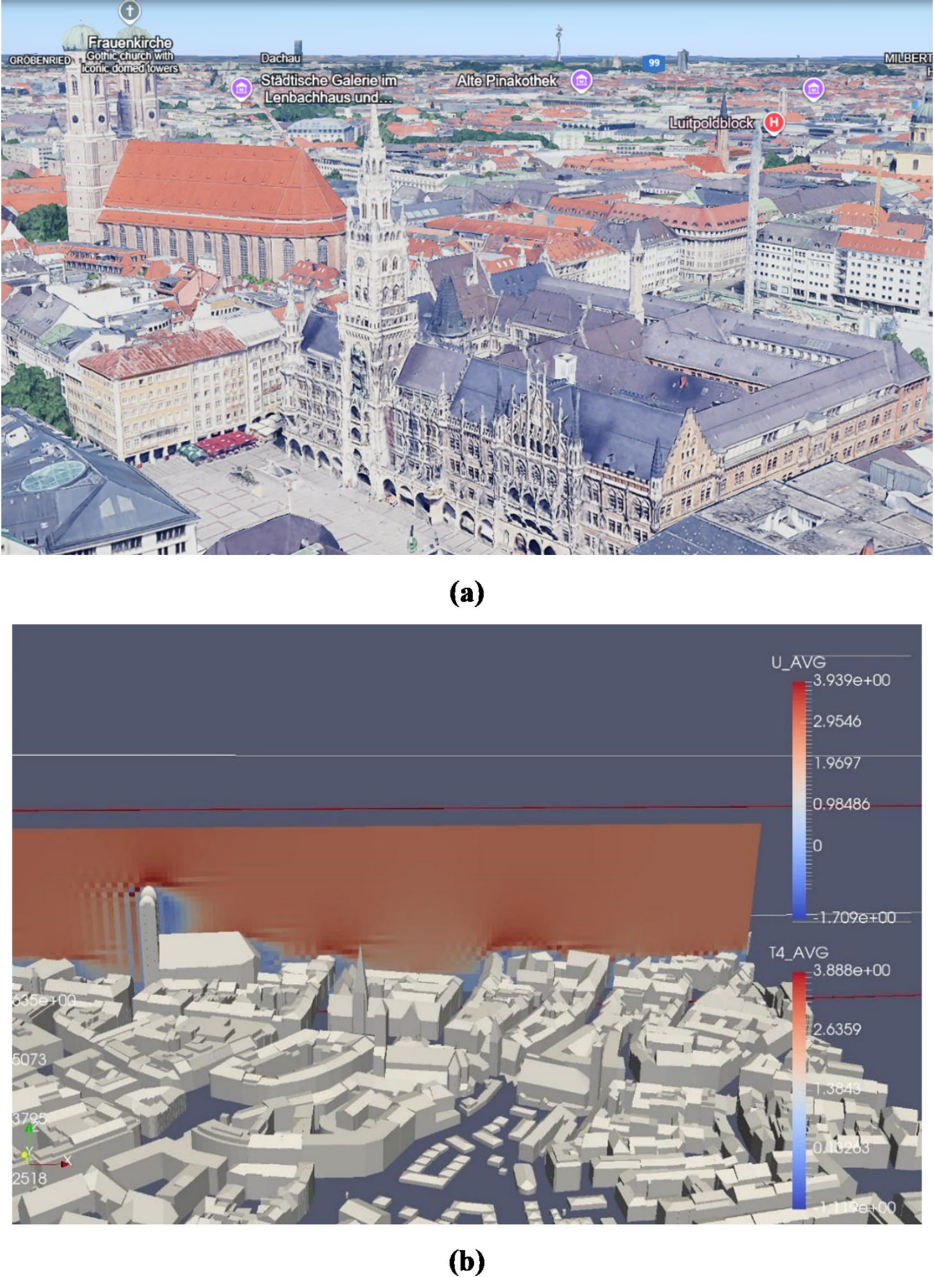
Velocity field in the vicinity of Frauenkirche (Imagery sourced from Google earth view, https://www.google.com/earth/ and MGLET source code visualized in ParaView-5.13.0). (a) Frauenkirche and Marienplatz area (Imagery sourced from Google earth view, https://www.google.com/earth/). (b) Checker-board patterns in front of Frauenkirche (Test data of Munich urban terrain as STL bodies from MGLET source code).

Checker-board-like patterns for velocity fields were seen in front of the buildings. One of these patterns is significantly observant in front of the iconic Frauenkirche (see Fig. 5). The cause of the problem was speculated to be the issue with the high cell peclet number. It was assumed that the problem would reduce/go away once the simulation was run in a finer grid resolution.

#### Compressibility effect

**Fig. 6:**
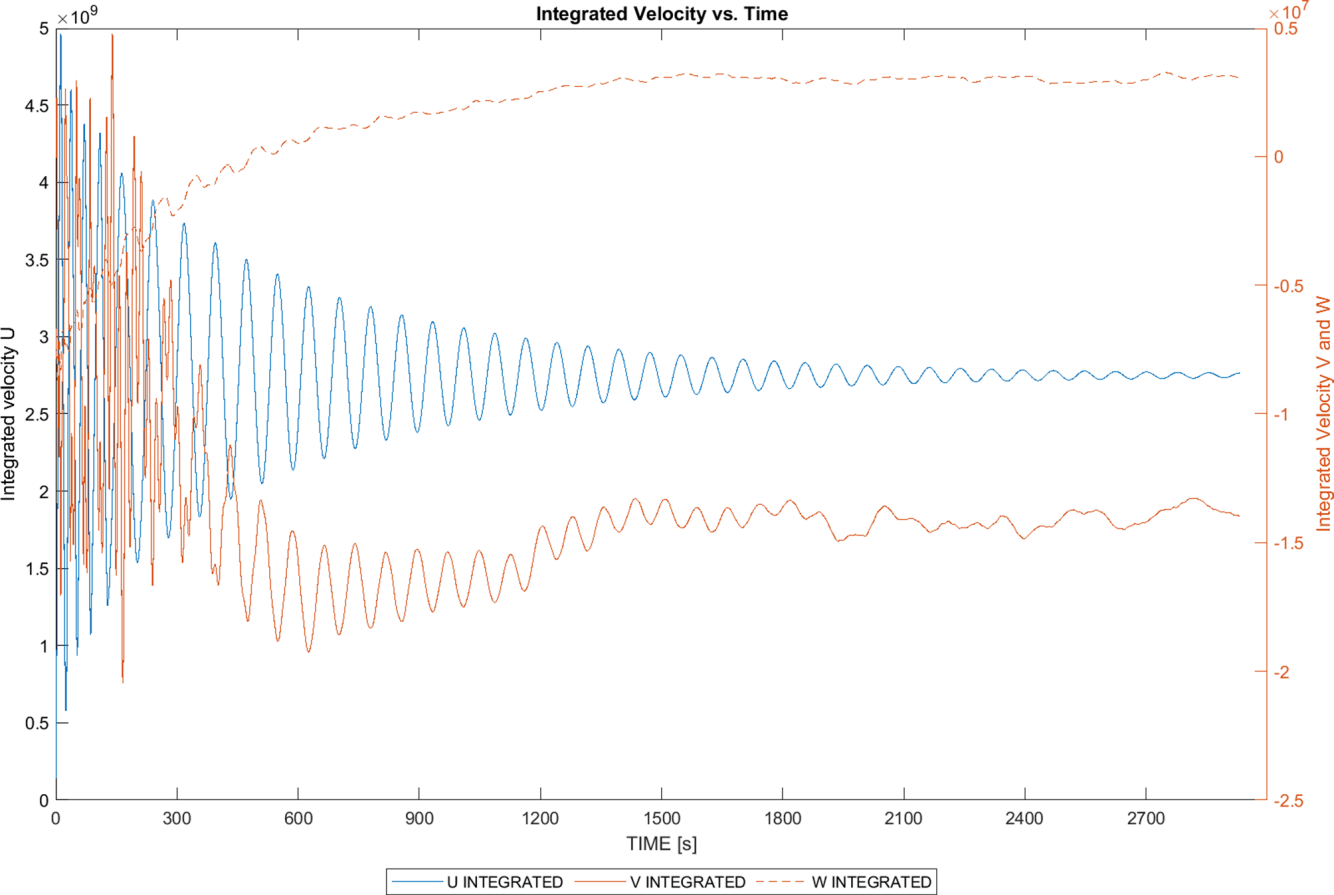
Development of integrated velocities for coarser grid resolution (graphs generated in MATLAB R2024a).

It was observed that, despite using the incompressible Navier-Stokes equations in the MGLET solver, the air inside the domain exhibited behaviour similar to that of a compressible fluid. This phenomenon is illustrated in Fig. 6, which displays a sinusoidal pattern in the evolution of integrated velocities over time. Although the sinuosity diminishes with time, it represents an unphysical phenomenon. The origin of this effect was attributed to the residual error encountered during the solution of the mass conservation equation. The variable EPCORR governs the number of iterations required to solve the equation. In this simulation, EPCORR was set to 5.0 × 10^− 3^, indicating that the pressure solver’s correction would iterate until the residual of the equation reached a value below this threshold. To mitigate this effect, one could simply reduce the value of EPCORR by several orders of magnitude, thereby encouraging a more divergent flow field.

#### Scalar development

**Fig. 7:**
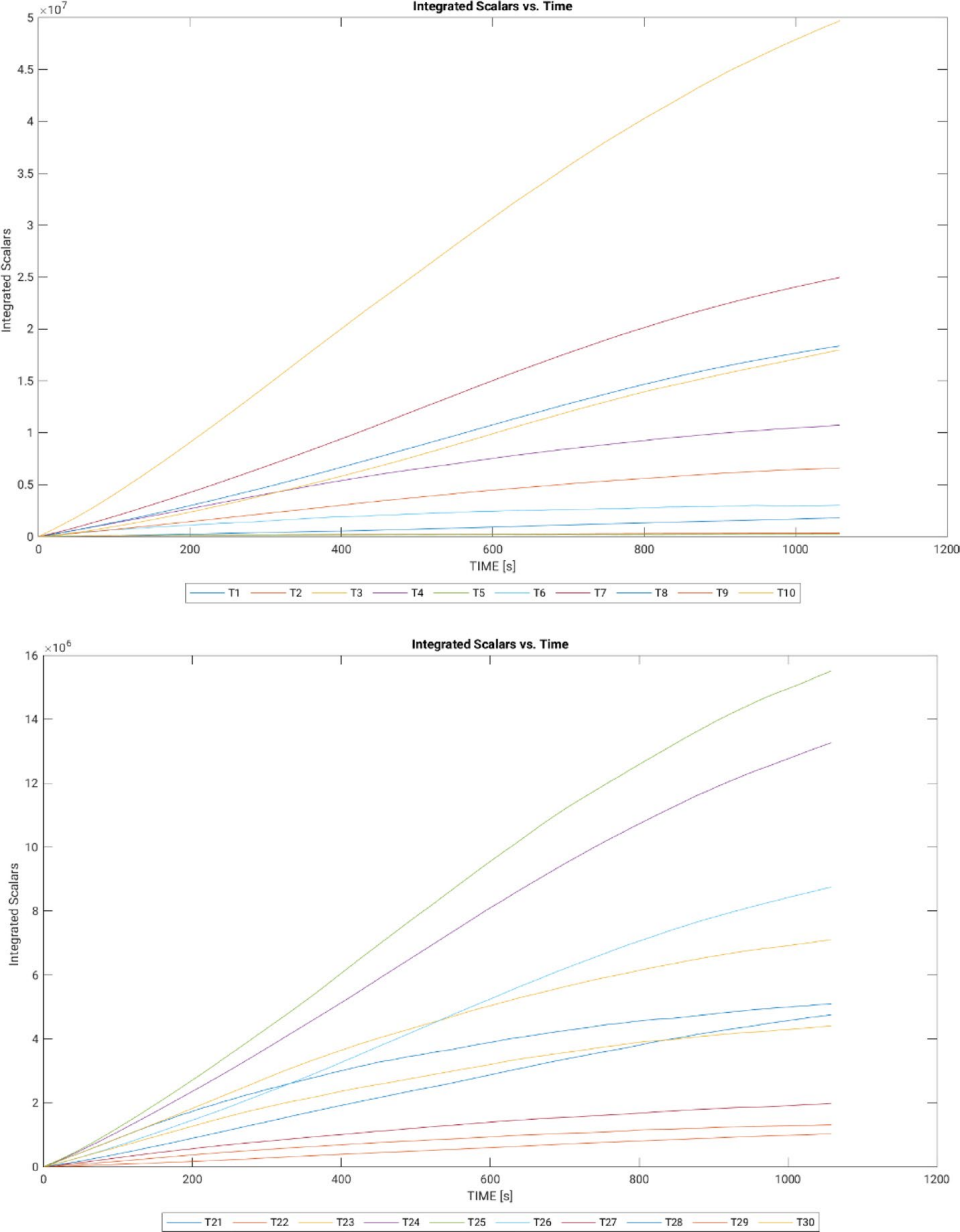

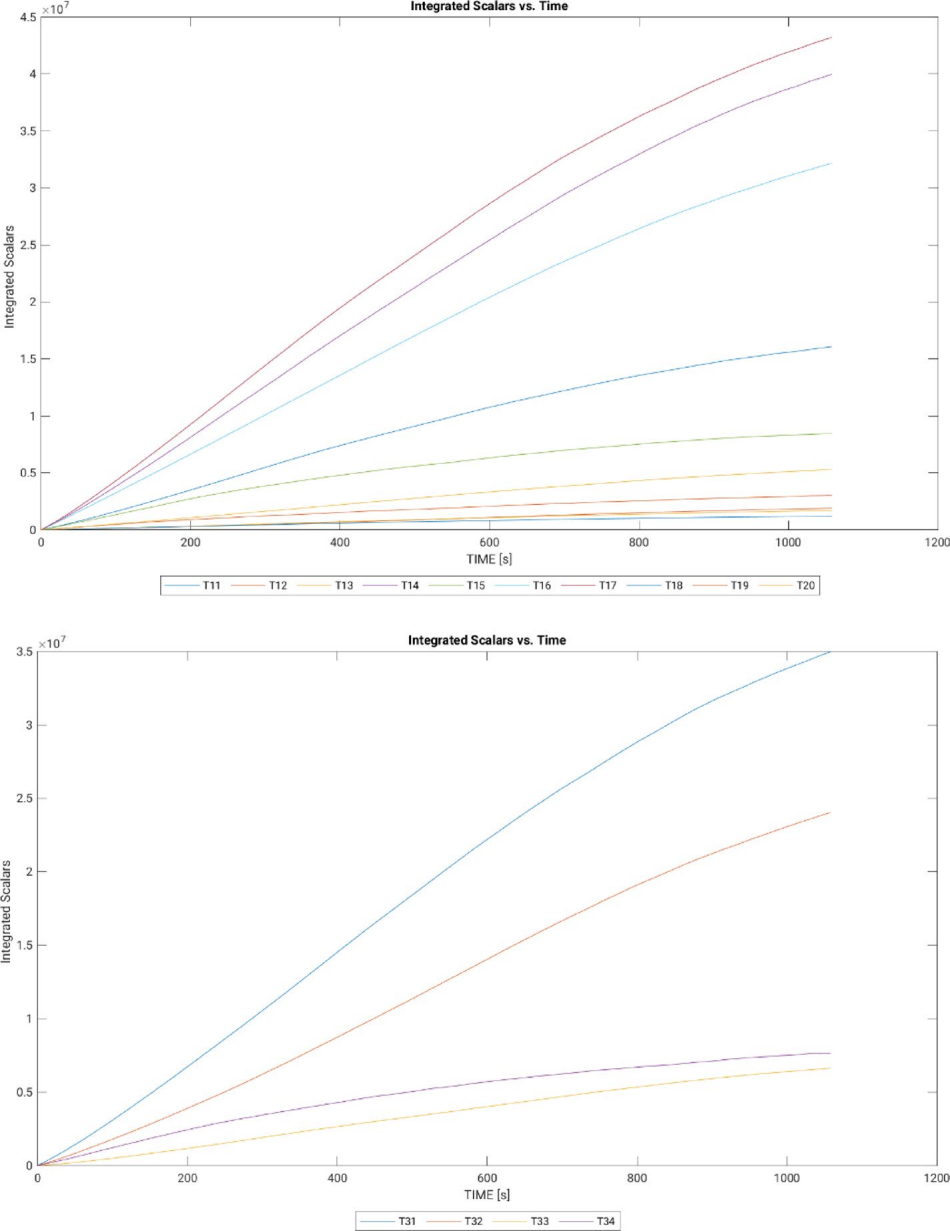
Development of scalars over time after bug patch (graphs generated in MATLAB R2024a).

The graphs below (see Fig. 7) present the integrated scalar progression for each street, with the linear trend indicating efficient code development. This supports the refinement of grids for higher resolution and enhances the analysis of wind-driven traffic pollutant dispersion.

### Simulation in finer grid resolution

After testing the new patch of MGLET on the coarse grid, we tested it on the fine grid. The new grid was specifically designed to achieve the finest refinement with an approximate length of 2.5 m in all dimensions. The refinement regions were strategically placed to the west of the Karslplatz Stachus area. The meshing of the domain, along with the refinement regions, is illustrated in Fig. 3.

#### Addressing problems

**Fig. 8:**
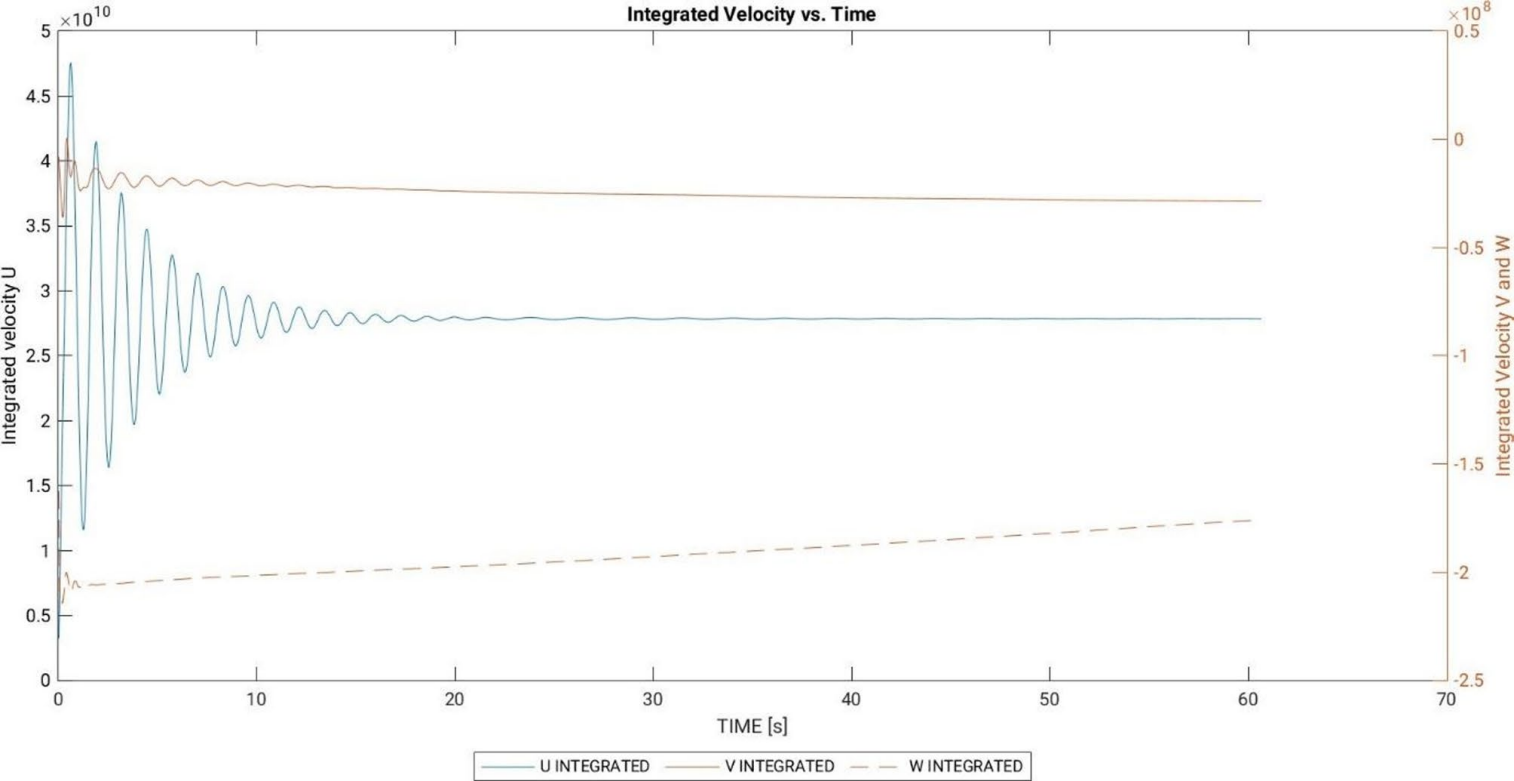
Development of integrated velocities for finer grid resolution (graphs generated in MATLAB R2024a).

The simulation on the fine grid was initially carried out on the local server, serving as a pre-cursor simulation before the main simulation. In this initial simulation, EPCORR was reduced from 5 × 10^− 3^ to 5 × 10^− 4^ to address compressibility effects, as discussed in Sect. 5.1.2. The effectiveness of this approach is highlighted in Fig. 8, where the integrated values of U, V, and W no longer exhibit a sinusoidal effect after some time and reach a steady state. Up to this point, the flow field was considered to be in a transient state, and statistics of velocity and scalar fields were not averaged.

#### Analysis of scalar field

The simulation was recreated for a heavy traffic duration on a Monday, 14th September 2020, from 10:00 to 11:00. The wind is documented to be 2.8 m/s at 270 degrees (west to east), which matches the $\:{vb}_{sensor}$ arranged in the wind inflow profile discussed in Sect. 4.4. Keeping this in view, the post-processing steps are carried forward from the simulation output.

##### Influential streets

The simulation yielded a set of 34 distinct scalars corresponding to individual streets. Focusing on our designated area of interest, Karlspatz Stachus, for more effective analysis, it became imperative to identify and isolate the scalars with significant contributions in the Karlsplatz Stachus vicinity. Consequently, we defined a rough volume of interest (VOI) near the area and examined the thresholds of the 34 scalars within this VOI. From the pool of 34 scalars, we identified and retained the top 10 scalars with the highest thresholds for further investigation. Table 3 provides a listing of these selected streets in descending order of their upper thresholds, along with the corresponding scalar thresholds.

**Table 3:** Influencing streets towards Karlsplatz stachus air quality station.

Number	Street	Lower threshold	Upper threshold
1	Karlsplatz	−3.54523682594299	186.301
2	Bayerstrasse	−0.000772697094362229	5.61269187927246
3	Landbergerstrasse	−0.00253034615889192	0.85065758228302
4	Luisenstrasse	0	0.32
5	PaulHeyse-Strasse	−0.00026107463054359	0.161
6	Paulheisseunterfhrung	0	0.120447635650635
7	Trappentreustrasse	0	0.087
8	MartinGreifStrasse	0	0.0763
9	Bavariaring	−0.000684100261423737	0.0203709416091442
10	Theresienhoehe	−0.00010922633373411	0.00323030631989241

Moreover, Fig. 2 visually depicts these streets and the chosen VOI for analysis, represented by the blue cuboid in the top right corner. Several instances of negative values in the lower threshold of the scalars can be observed, which pose a physical impossibility. Such negative values are likely attributable to numerical errors during the simulation process.

##### Emission weights

We assigned unit emissions from each street to the corresponding scalar in the source code. However, in reality, different streets have different rates of emission. We approached the weight-based system for the influential streets to have this realistic scenario. The weights were defined using traffic volume flowing through each street. We obtained the traffic volume map of 2022 for the city of Munich based on two vehicle categories, light and heavy-duty traffic from the Munich Mobility Department. Since the simulation already accounts for the street length, individual street concentrations in our calculation did not consider this factor. The traffic count for the city of Munich for two classes of vehicles, lightweight and heavy traffic, is obtained from traffic volume maps. Additionally, several key assumptions are adopted for this model:

- Only two types of vehicles (Passenger cars, Diesel Euro 6d and Heavy Duty Euro IV), each with the same characteristics.
- Energy consumption of 1.17 kWh per kilometre for heavy-duty vehicles.
- All vehicles operate at similar velocities.
- All vehicles are used in a similar manner for acceleration and braking.
- Emissions due to traffic share have not changed significantly in recent years.

In order to conceptualize the individual contribution of each street, normalized weights were calculated based on their associated traffic load. The concentration of NOx emissions in grams per day can be estimated by following the recommendations of the European Union emission regulations. This involves adopting factors corresponding to category M - Passenger Cars, Euro 6d Regulation, and Euro VI for heavy-duty vehicles. The formula for calculating NOx emissions is given by:

$${\text{NOx }}\left( {{\text{g}}/{\text{day}}} \right)\,=\,{\text{Street Network }}\left( {{\text{Km}}} \right) \times {\text{Traffic }}\left( {{\text{pKW}}/{\text{day}}} \right) \times {\text{Regulation factors}}$$

Utilizing traffic count data and regulatory factors, we computed normalized weights for emissions associated with influential streets, as outlined in Table 4. Streets experiencing higher traffic counts tend to exhibit increased emission weights.

**Table 4:** Normal and heavy traffic counts for influential streets with normalized weights for emissions.

Street name	Scalar	Normal traffic	Heavy traffic	Weights	Normalized weights
Karlsplatz	T15	44	2	4.5964	10.69%
Bayerstrasse	T4	9.5	0.55	1.05601	2.46%
Landbergerstrasse	T16	24.857	1.35	2.71513	6.32%
Luisenstrasse	T18	13	0	1.04	2.42%
PaulHeyse-Strasse	T23	21	0.3	1.84146	4.28%
Paulheisseunterfhrung	T22	21	0.7	2.05674	4.79%
Trappentreustrasse	T32	34.2	1.1	3.32802	7.74%
MartinGreifStrasse	T20	113	4.8	11.62336	27.04%
Bavariaring	T3	14.5	1	1.6982	3.95%
Theresienhoehe	T31	124.5	5.7	13.02774	30.31%

##### Volume averaged total scalar

**Fig. 9:**
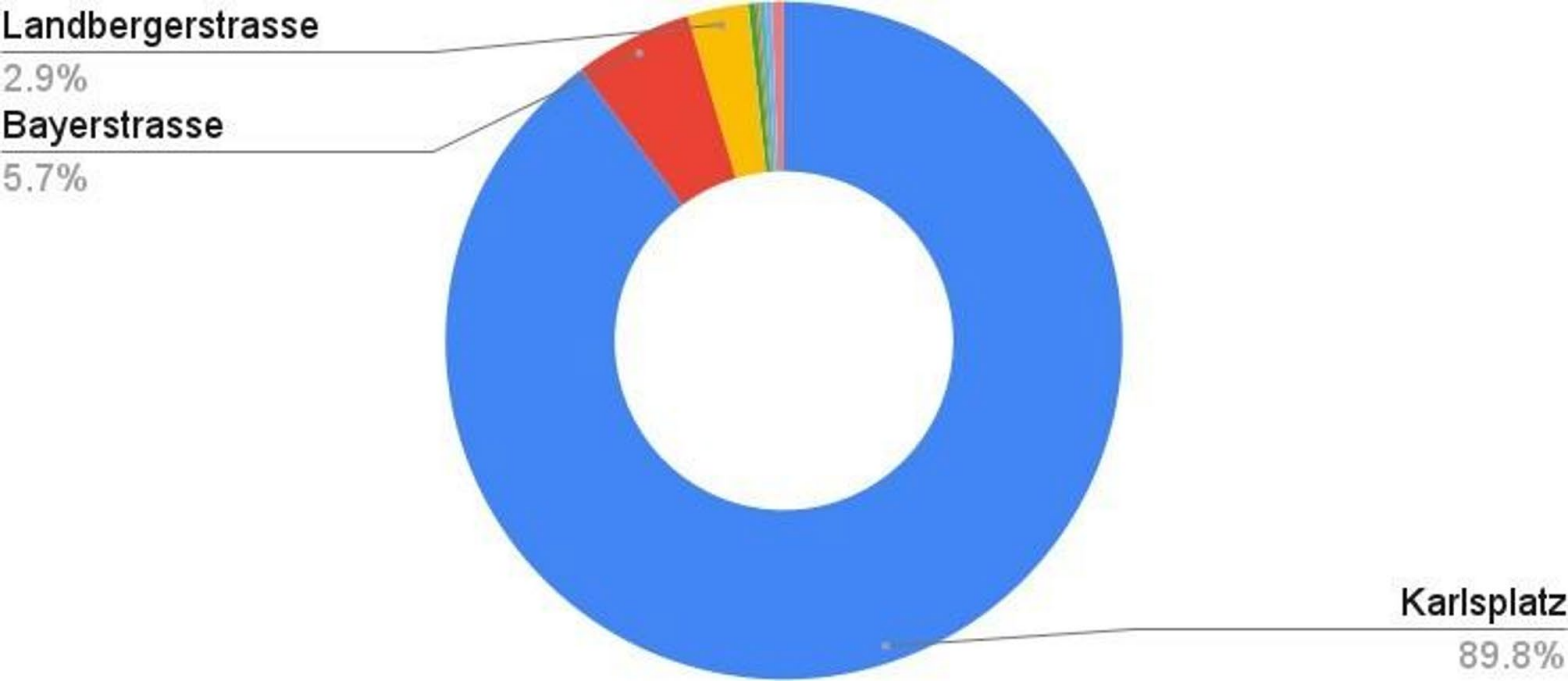
Contribution of streets for pollutant concentration at Karlsplatz Stachus. (Source: Authors)

To quantify the average total scalar present near Karlsplatz Stachus air quality station, a 40 × 40 × 10 m VOI was defined. The approximate location of the VOI was selected based on the air quality index map^46^. All the quantities were clipped to this VOI, and their attributes were appended together for further analysis. First, the scalars from influential streets were integrated over this VOI and averaged to create volume-averaged scalars. Then, the volume-averaged scalars were multiplied by the weights evaluated in Table 4. The weighted volume-averaged scalars were finally normalized to evaluate the actual influence of emission from each street to the air quality measuring station. The evaluation of normalized and weighted volume Averaged total Scalar is presented in Table 5. The results show that traffic from Karlsplatz Street is responsible for 89.75% of pollutant concentration at the measuring station followed by Bayerstrasse and Landbergstrasse with 5.65% and 2.90% respectively. These contributions can also be visualized in Fig. 9.

**Table 5:** Evaluation of normalized and weighted volume averaged total scalar.

Street name	Weights	Volume averaged scalar (VAS)	Weighted VAS	Normalized VAS
Karlsplatz	10.69%	13.675	1.462	89.75%
Bayerstrasse	2.46%	3.749	0.092	5.65%
Landbergerstrasse	6.32%	0.748	0.047	2.90%
Luisenstrasse	2.42%	0.239	0.006	0.35%
PaulHeyse-Strasse	4.28%	0.060	0.003	0.16%
Paulheisseunterfhrung	4.79%	0.107	0.005	0.31%
Trappentreustrasse	7.74%	0.063	0.005	0.30%
MartinGreifStrasse	27.04%	0.034	0.009	0.56%
Bavariaring	3.95%	0.002	0.000	0.01%
Theresienhoehe	30.31%	0.000	0.000	0.01%

Moreover, the recorded concentration of NO_x_ at the measuring station, corresponding to the date and time of our scheduled simulation scenario, is 44 *µg/m*3. We aim to calibrate the CFD model such that the volume-averaged concentration in the aforementioned VOI matches the measured data. Using the weights from Table 4, we implemented the following mathematical expression using the calculator function with the VOI:

$$\begin{gathered} (10.69 \times T{15_{avg}}\,+\,2.46 \times T{4_{avg}}\,+\,6.32 \times T{16_{avg}}\, \hfill \\ +\,2.42 \times T{18_{avg}}\,+\,4.28 \times T{23_{avg}}\,+\,4.79 \hfill \\ \times T{22_{avg}}\,+\,7.74 \times T{32_{avg}}\,+\,27.04 \times T{20_{avg}} \hfill \\ \,+\,3.95 \times T{3_{avg}}\,+\,30.31 \times T{31_{avg}})/3.7022 \hfill \\ \end{gathered}$$

**Fig. 10:**
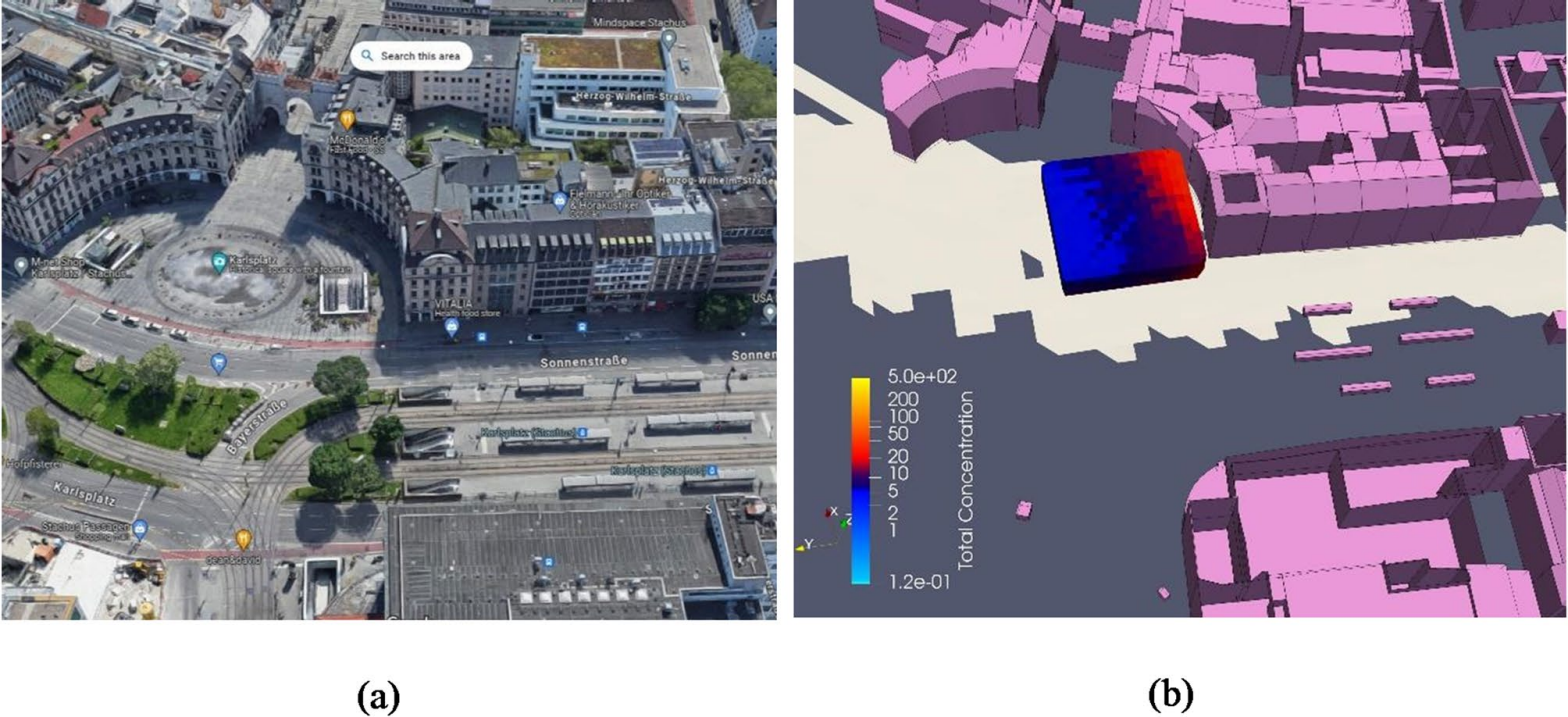
Comparison at Karlsplatz Stachus (simulation result on ParaView-5.13.0, https://www.paraview.org). (a) Google Earth view of Karlsplatz Stachus. (b) Distribution of scalar in the VOI.

The resultant volume-averaged concentration within the VOI has been determined to be 44. The denominator, represented by the numerical value 3.7022, serves as the calibration factor. Figure 10a provides a Google Earth view of the vicinity near Karlspplatz Stachus, while Fig. 10b illustrates the scalar distribution in the VOI based on the aforementioned expression. Notably, the concentration of total scalars is observed to be higher in proximity to buildings compared to the open areas. This observation aligns with the expectation that NOx may accumulate in regions near buildings.

**Fig. 11:**
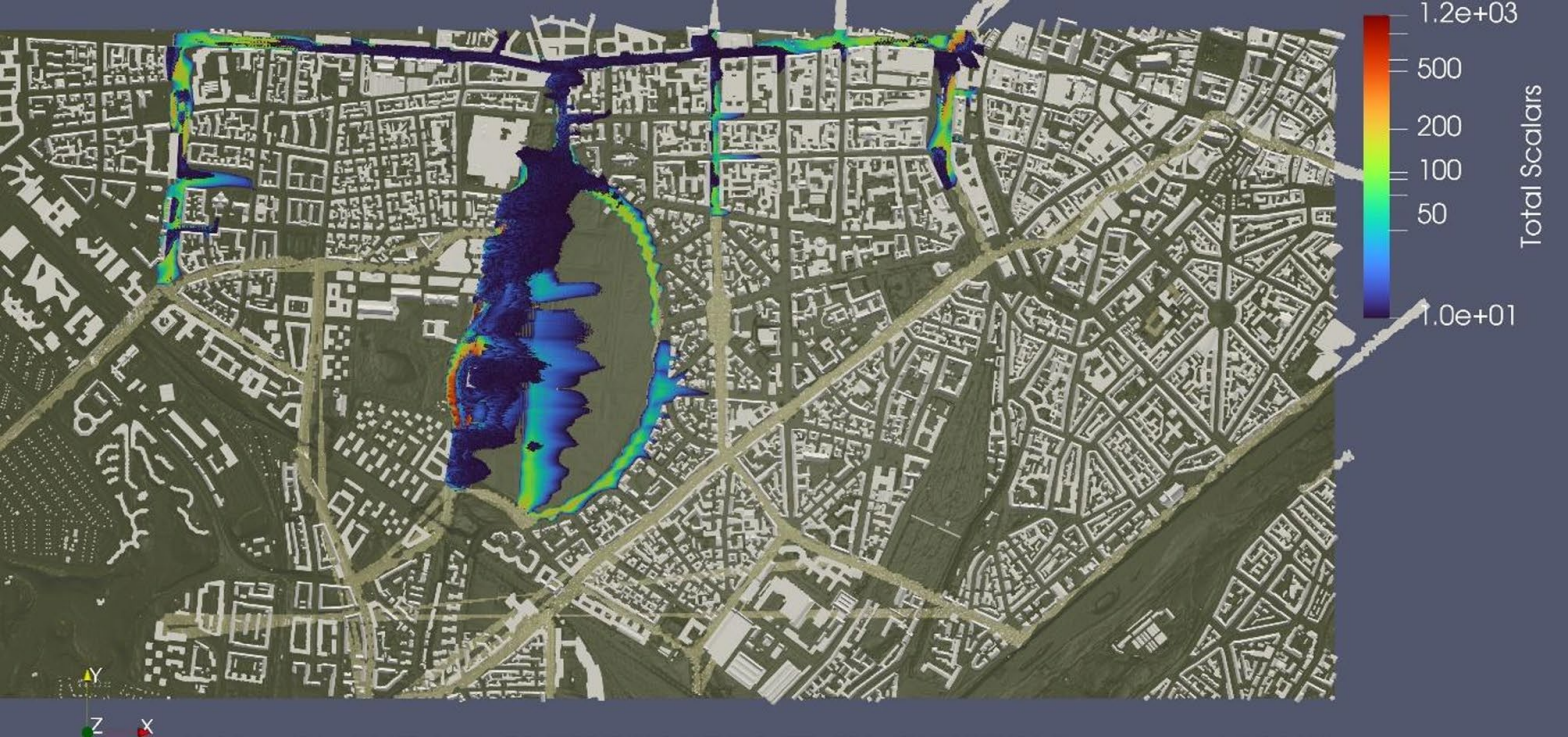
Distribution of weighted scalars from influential streets (visualization result on ParaView-5.13.0, https://www.paraview.org from simulation run on MGLET).

**Fig. 12:**
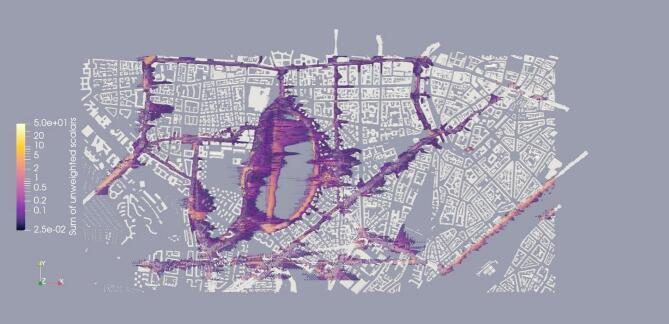
Distribution of unweighted scalars from all 34 streets (visualization result on ParaView-5.13.0, https://www.paraview.org from simulation run on MGLET).

Similarly, the distribution of traffic-weighted scalars from influential streets can be seen in Fig. 11. The figure clearly depicts how the scalars are spread east to the streets, which is to be expected since the wind is flowing from west to east. The scalars are also concentrated mostly near the streets and areas that are surrounded by buildings, which makes the transport of scalars difficult. Additionally, the distribution of unweighted scalars from all 34 streets can be seen in Fig. 12. A similar pattern of distribution of scalars is noticeable here as well.

##### Analysis of velocity fields

**Fig. 13:**
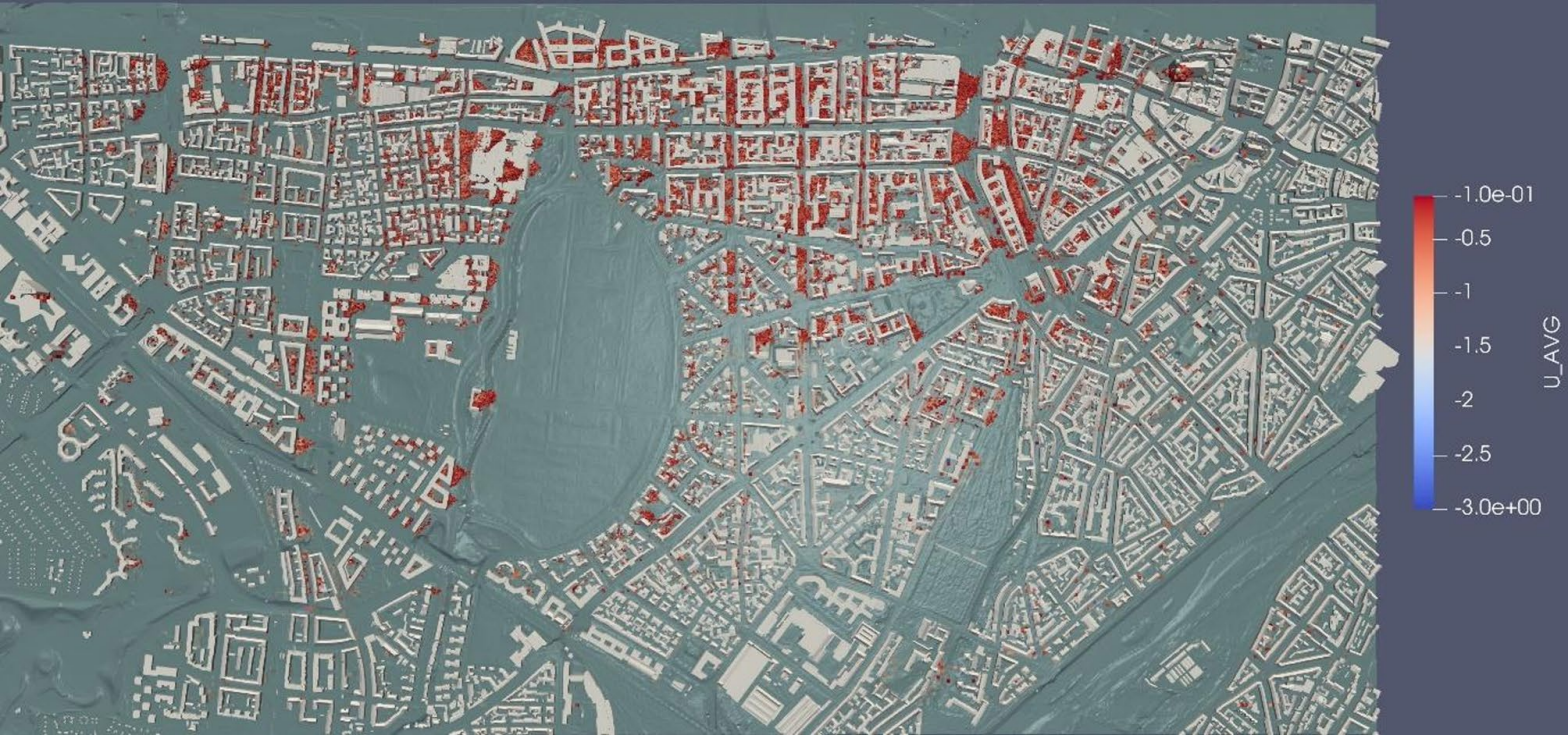
Recirculation zones highlighted by negative average U-velocity (visualization result on ParaView-5.13.0, https://www.paraview.org from simulation run on MGLET).

**Fig. 14:**
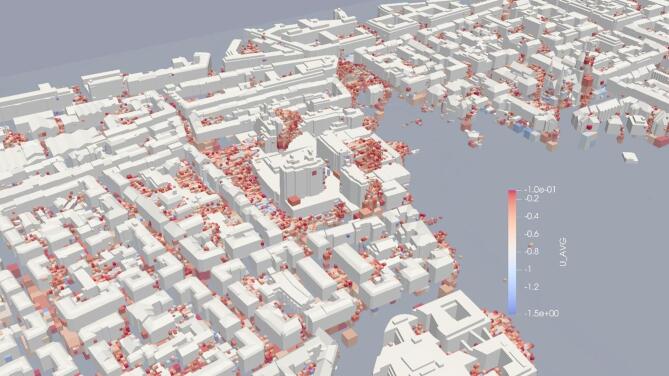
Close-up view of recirculation zones (visualization result on ParaView-5.13.0, https://www.paraview.org from simulation run on MGLET).

While the primary focus of this study was to analyse the transport of multiple pollutants, we came across some noteworthy observations in the velocity field. In Fig. 13, negative values in the average U-velocity fields behind the buildings (east side buildings) indicate wind flow recirculation in these areas. Recirculation zones are more pronounced behind buildings aligned with the wind flow, as opposed to those at oblique angles to the wind direction. A closer examination of these recirculation zones is provided in Fig. 14.

**Fig. 15:**
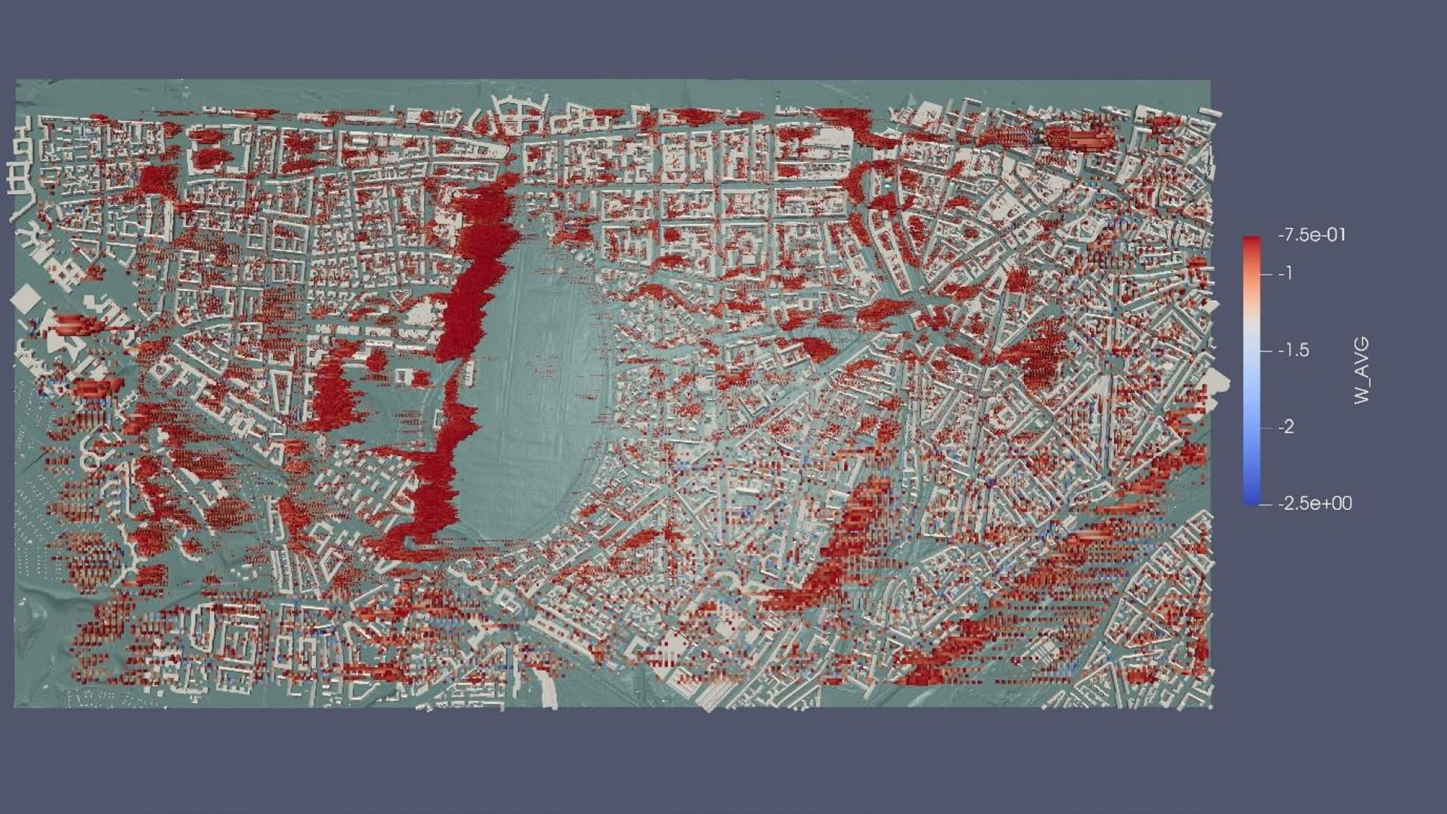
Areas with dominant W-velocity (visualization result on ParaView-5.13.0, https://www.paraview.org from simulation run on MGLET).

Likewise, Fig. 15 emphasizes areas with dominant W-velocity values, particularly in front of and behind buildings. Notably, the region near the Isar River (bottom right side of Fig. 15) also exhibits negative W-velocity-dominated areas. This phenomenon may be attributed to the abrupt change in terrain from an urban setting to a contoured floodplain.

## Limitations and outlooks

In this study, the analysis of observations was centred around a single measuring station. Consequently, the analysis and “conceptual calibration” of the CFD model lack direct validation. An improved approach would have involved incorporating two or more measuring stations within the domain. This multi-station setup would have allowed for cross-validation of emission weights and the calibration factor across multiple measuring stations, enhancing the robustness and reliability of our findings. Having additional measuring stations would provide a more comprehensive assessment of the model’s performance and increase confidence in the derived emission weights and calibration factor.

Additionally, a computational constraint of 250 million cells was imposed, leading to challenges in the velocity fields, even in the regions with the finest refinements, as highlighted in Table 2. While we opted for a domain height of 500 m, a more optimal approach might have involved using a domain with a smaller height and reallocating the available computational cells to refine influential areas.

There were also some obvious unphysical observations, such as negative scalar values, resulting from numerical error. Therefore, more attention might be needed to resolve these numerical errors by using higher-order schemes in the computation.

The domain in our study was limited to digital terrain and buildings, overlooking the potential impact of other elements, such as trees, on wind flow and scalar distribution. While it is acknowledged that incorporating trees and additional objects in a CFD approach can present challenges and may be considered unrealistic, there is room for future investigations to explore ways of accounting for these effects.

In subsequent research endeavours or in the event of a continued investigation by subsequent scholars, it would be advantageous to seek remedies for the identified limitations. Such efforts have the potential to substantially enhance both the modelling precision and efficiency. Furthermore, the model’s robustness can be fortified by incorporating additional meteorological parameters in the future. These parameters may encompass heat transfer, pollutants from sources other than vehicles, and other relevant factors. The absorption of contaminants will also be a crucial aspect to address in order to make the model more realistic.

## Conclusion

Hence, it is clear that realistic models pertaining to atmospheric boundary layers can be crafted with a notable level of qualitative precision. Progressing with the geodata model to achieve a comprehensive representation of terrain, along with integrating the wind profile of urban terrain and applying boundary conditions, marks a favourable step in model development. The obtained simulated emission data, coupled with the comparison of scalar emission and traffic load, validates this approach to model design and simulation. Nevertheless, there is room for further refinement, particularly in comparing the obtained emissions with the station data values. This aspect can then be addressed by relevant agencies, such as environmental bodies, for their respective purposes.

## Data Availability

The datasets used and analysed during the current study available from the corresponding author on reasonable request.
